# Supplementary material for: Are carbon emissions trading and green financial instruments synergistic? -Comprehensive quantitative research based on content analysis
Source: PLoS One. 2024 Mar 7;19(3):e0298601. doi: 10.1371/journal.pone.0298601 (PMC10919601; doi:10.1371/journal.pone.0298601)
Supplement: S1 Data — (ZIP) [file pone.0298601.s001.zip › Data/地级市Z_F工作报告.zip!/╕ú╜¿/╞╬╠∩╩╨/└┤╫╘╣1⁄2╓┌║┼í╛┬φ┐╦╩2╛▌═°í┐_2009.docx]

各位代表：

　　现在，我代表莆田市人民政府向大会报告工作，请予审议，并请市政协各位委员和其他列席同志提出意见。

一、积极应对 奋发有为 湄洲湾港口城市发展取得新成就

　　2008年是很不平凡的一年，我们经历了重大考验和严峻挑战。在中共福建省委、省政府和中共莆田市委的正确领导下，全市人民积极应对，奋发有为，深入贯彻落实科学发展观，按照“四求先行”、“四个重在”和“四用发展”要求，全面落实市委五届五次、六次全会的部署，大力实施“以港兴市、工业强市”发展战略，扎实推进湄洲湾港口城市建设，经济社会发展取得了新成就。

　　一是经济实力持续增强。初步核算，全市生产总值609.96亿元，增长15%。财政总收入52.78亿元，增长15.6%，其中地方级财政收入29.56亿元，增长24.1%。全社会固定资产投资299.58亿元，增长24.3 %。外贸进出口总额23.2亿美元，增长8%，其中出口总额17.2亿美元，增长10.8%。实际利用外资(历史可比口径)5.63亿美元，增长1.7%。合同利用外资(历史可比口径)4.07亿美元。社会消费品零售总额206.1亿元，增长19.3%。城镇居民人均可支配收入16495元，实际增长10.7%。农民人均纯收入6436元，实际增长10%。居民消费价格指数104.9%。城镇登记失业率2.32%。产业结构不断调整，三产比例为12.3∶56.5∶31.2。农业稳步发展，预计农林牧渔业总产值124.9亿元，增长5.2%。粮食总产量30.3万吨，肉蛋奶、水产品产量持续增长。农业产业化加快推进，新增国家级龙头企业1家、省级龙头企业3家，市级以上龙头企业带动农户55万户，实现产值95亿元。工业加快发展，实现增加值300.1亿元，增长19.1%。规模工业企业经济效益综合指数187.3%，提高7.1个百分点，规模工业企业利润17.2亿元。新增规模以上工业企业210家，总数达到1020家。福建LNG站线工程和莆田燃气电厂1号机组、海安巨型工程轮胎、海宏木业、万邦电子、光纤陶瓷插芯一期、恒信塑胶等重点工业项目建成投产。林浆纸一体化、光电伏特、国航造修船基地、兴化湾南岸综合开发、石门澳临港产业园、南日岛风电场三期、湄洲湾火电厂二期、莆田核电站等生产性项目前期工作加快推进。服务业继续发展，实现增加值190亿元，增长11.9%。工艺美术城全面建成并投入使用。家乐福、永辉超市落户莆田。银行、证券、保险业服务保障能力增强，年末金融机构人民币存款余额比上年末增长18.9%，贷款余额比上年末增长17.6%。旅游总收入增长18.8 %。品牌战略持续推进，新增中国驰名商标3件、国家出口免验产品1个、福建省著名商标12件、福建省名牌产品29项，获得福建省品牌农业企业金奖1个，制定企业技术标准国家级1个、省级3个，荣获“中国鞋业出口基地”、“中国银饰之乡”、“中国枇杷之乡”称号，莆田枇杷、桂圆获得“国家地理标志产品保护”称号。节能减排工作取得成效，单位生产总值能耗实现年度目标，二氧化硫排放量和化学需氧量进一步削减，预计万元规模工业增加值能耗为0.45吨标准煤，下降22.7%。成功入选第四届华商大会评选的“2008中国城市投资吸引力百强城市”，入选福布斯“中国大陆最佳商业城市榜”。

　　二是重点建设有力推进。227个重点项目完成年度投资152.2亿元，基础设施投资54.8亿元，分别占全社会固定资产投资的50.8%和17.7%。港口交通体系加快建设，LNG10万吨级专用码头、秀屿4万吨级木材专用码头投入生产，湄洲湾深水航道一期工程完工，东吴作业区8个深水泊位抓紧施工，罗屿25万吨矿石码头等港航项目前期工作进展顺利，港口货物吞吐量达到1802万吨。福厦铁路莆田段、向莆铁路莆田段加快建设，湄洲湾港口铁路支线开工建设。省道202线荔城改线段、荔港大道主车道、东园路基本建成，城港大道一期、沁峤路一期、仙港大道基本贯通。福泉高速公路莆田段扩建工程、莆秀高速公路、省道201线建设进展顺利。湄永高速公路莆田段、福广高速公路莆田段、湄洲岛跨海通道、莆田机场等交通项目前期工作加快推进。LNG新增储罐顺利开工。LNG冷能产业园、石城－石井风电、220千伏主干电网、35千伏及以上输变电等能源项目加快建设，全市电力装机容量达到124万千瓦。金钟水利枢纽、木兰溪霞林段防洪工程、北洋水系整治二期、东圳灌区节水改造等水利项目加快推进。英博雪津啤酒脱硫工程建成运行，湄洲湾电厂一期烟气脱硫工程、闽中污水处理厂配套管网等环保项目加快建设。

　　三是城乡发展更加协调。完成新一轮城市总体规划修编纲要和妈祖城核心区等65项城市规划设计，抓紧编制兴化湾南岸发展规划，做好乡（镇）村规划，优化城乡布局。实施城建项目96个，总投资89.2亿元，完成年度投资11.4亿元。滨海新城加快建设，妈祖城一期填海造地2631亩，中铁公司到资2亿元并投入建设。妈祖阁顺利落成。城区路网不断完善，新增城市道路30公里。国家园林城市创建工作扎实推进，城市绿化、亮化、美化工程加快实施，新增绿地面积125公顷，建成区绿化覆盖率达到39.1%。城市污水处理率、垃圾无害化处理率分别达到73.6%、83.7%。旧城改造步伐加快，拆除旧房建筑面积83.6万平方米，建设各类安置房87.3万平方米，回迁群众1320户。生态市建设有力推进，生态公益林面积不断扩大，森林覆盖率达到56.3%。耕地保护成效明显，连续9年实现耕地总量动态占补平衡，建立建设用地监管和国土资源执法长效机制，清理工业闲置土地125宗6088亩，新审批建设用地23075亩。100个新农村试点村加快建设，建成一批省级村镇住宅试点小区。农业面源污染有效治理，在500个行政村实施“家园清洁行动”，农村生活垃圾无害化处理率达到75%以上。建成一批农村水利、道路、电力、通讯等基础设施，加固海堤13.5公里，除险加固水库24座，新增农村沼气5850口、农村客运站（亭）332个、农村水泥公路320公里、陆岛交通码头5座，改造危桥40座，在全省率先实现村村通水泥路、村村通客运班车、岛岛有交通码头目标。完成电力、移动电话、广播电视“村村通”工程年度任务，864个行政村（社区）接通互联网宽带。饮水安全村村通工程有效推进，受益 78.9万人。11件为民办实事项目基本完成。

　　四是改革开放不断深化。集体林权、土地承包经营权、海域使用权等改革取得实效。国有资产监管力度加大，公共资源市场化配置取得初步成效。代建制试点工作稳步推行。企业改制上市步伐加快，郭氏、才子、三棵树、东南香等22家企业启动改制上市有关工作。自主创新能力不断提升，建成国家浆纸质检中心、省贵重金属和珠宝玉石质检中心，新增3个省级企业技术中心、12个市级企业技术中心。第六届中国海峡项目成果交易会对接项目626个。获得专利公开公告221项，列入国家和省科技项目计划26个。对外交流进一步扩大，成功举办莆田（广州、彭州）招商项目推介会、第三届中国（莆田）海峡工艺品博览会、第三届莆田国际制鞋技术设备？鞋材展览会，积极参加海峡两岸经贸交易会、中国福建商品交易会、中国国际投资贸易洽谈会等各种招商推介会。与美国贝茨维尔市、加拿大坎伯兰市建立友好城市关系。莆台经贸文化交流更加密切，秀屿港（含湄洲岛）被列为国家首批两岸直接“三通”口岸。第十届妈祖文化旅游节成功举办。

五是社会事业有效提升。创新型城市建设步伐加快，“2008院士专家海西行暨人才？科技项目对接会”顺利举办。城乡教育发展更加均衡，农村办学条件不断改善。“双高普九”成果巩固提高，农村义务教育“两免一补”政策全面落实，农村中小学危房改造6万平方米，新建校舍7.7万平方米。莆田一中妈祖城校区等一批学校加快建设。普通高考再创佳绩，本科录取率达到32.3％。湄洲湾职业技术学院顺利通过教育部人才培养评估。体育事业蓬勃发展，第十四届省运会筹备工作有序推进，莆籍运动员获得2008年北京奥运会特设项目比赛金牌1枚。举办央视“2008迎中秋大型民族音乐会”、第二届莆田文学节。评选“莆田十大城市名片”。“妈祖信俗”被文化部推荐申报世界人类非物质文化遗产。第二届《莆田市志》编纂任务基本完成。中华妈祖文化研究院和莆仙大剧院主体工程顺利落成。医疗救治体系逐步完善，爱国卫生运动深入开展，有效防治登革热、手足口病等疫情疫病。新建和改扩建19所乡镇卫生院。强化“食品放心工程”建设，深入开展食品药品安全专项整治。群众性精神文明创建活动深入开展。人事、编制、气象、防震、海事、保密、档案、科普、工会、共青团、妇女儿童等工作继续加强。

　　六是和谐社会稳步构建。中央和省各项强农惠农政策有效落实，市级以上财政用于农村各项投入达到12亿元。新型农村合作医疗参合率达到94.1%，医疗救助农村困难家庭人员9.2万人。为1200多名贫困白内障患者免费施行复明手术，为371名肢残患者实施助行。城镇新增就业2.3万人，下岗再就业2047人，农村劳动力转移就业4万人。居民最低生活保障标准进一步提高，农村低保受益群众86792人，城市低保受益群众13213人。实施库区建设项目59个，发放库区移民后期扶持直补资金2058.5万元。“造福工程”搬迁3700人。人口与计划生育工作得到加强，人口自然增长率6.2‰，完成省下达的人口责任指标。发放住房公积金贷款2.27亿元，惠及1009户家庭。建成赤港“侨居造福工程”168套2.1万平方米。改建修缮贫困残疾人危房，惠及200户877人。建设廉租房7400平方米，1580户城市低保家庭住房困难得到解决。举办央视“春暖2008中国（莆田）工艺珍藏品爱心拍卖晚会”，建设博爱卫生院（所）29所。为四川地震灾区捐款捐物1.07亿元，对口支援濛阳镇灾后重建。双拥共建持续深入，第三次荣膺“全国双拥模范城”称号。“平安莆田”建设得到加强，群众对社会治安总体满意率达到96.7%，我市在全省社会治安综合治理年度考评中从2007年的第8名跃升为第4名。“五五”普法深入开展，为困难群众实施法律援助920件。安全生产责任制进一步落实，安全生产形势持续平稳。民族、宗教、老区、老龄等工作取得新成绩。国防动员、民兵预备役、海防、人防等工作取得新进展。

　　在过去的一年，我们努力建设服务型政府，自觉接受市人大及其常委会的法律监督、工作监督和政协的民主监督；认真办理人大代表议案、建议和政协提案。重视群众来信来访，积极化解社会矛盾。深化机关效能建设，推进政府信息公开，清理行政许可事项，规范行政自由裁量权。加强电子政务系统和政府网站建设，建立网上审批和电子监察系统，继续推进“数字莆田”建设。严格审计监督，强化廉政建设，严肃查处各类腐败案件。

　　各位代表，2008年，宏观形势严峻复杂，我们能取得上述成绩，实属不易。这得益于中共莆田市委的坚强领导，得益于全市人民的团结奋斗，得益于改革开放30周年的成功实践。在此，我谨代表莆田市人民政府，向各位代表和委员，向全市各行各业的劳动者和离退休老同志，向各民主党派、工商联、各人民团体和社会各界人士，向驻莆部队官兵、武警指战员以及所有关心支持莆田建设发展的台港澳同胞、海外侨胞、国内外朋友，表示崇高的敬意和衷心的感谢！

　　在看到成绩的同时，我们也清醒地认识到，我市经济社会还面临不少困难和问题，主要是：受世界金融危机和宏观经济环境影响，全市生产总值、固定资产投资、社会消费品零售总额等经济指标未能完成预期计划；经济总量仍然较小，人均指标与全省平均水平差距较大，综合实力不强；经济结构性矛盾突出，产业集聚效应不明显，工业骨干企业偏少，部分企业生产经营困难，现代服务业比重较低；外贸出口形势严峻，实际利用外资没有明显突破；资源环境约束加剧，节能减排形势不容乐观；城乡二元结构矛盾突出，区域发展不平衡，城乡居民收入差距较大；农业基础设施薄弱，农资价格波动较大，农民持续增收难度加大；社会保障水平较低，教育、文化、就业、医疗、社会治安等关系群众切身利益的问题仍然较多，部分低收入群众生活比较困难；安全生产形势仍然严峻，食品药品专项治理存在薄弱环节；政府职能转变有待加强，部分机关工作人员办事效率不高，存在形式主义、官僚主义现象，甚至发生了一些违法违纪案件等等。我们要高度重视这些问题，采取有效措施认真加以解决。

二、坚定信心 持续运作 全力推进经济社会又好又快发展

　　2009年是新中国成立60周年，也是加快建设湄洲湾港口城市的重要一年。当前，国际金融危机持续蔓延带来前所未有的挑战，国家扩大内需促进经济增长的宏观环境也带来前所未有的机遇。我们要审时度势抢机遇，坚定信心促发展，持续运作抓落实，奋发有为创大业，确保经济社会再上新台阶。

　　政府工作的总体要求是：全面贯彻落实党的十七大、十七届三中全会精神，高举中国特色社会主义伟大旗帜，以邓小平理论和“三个代表”重要思想为指导，深入贯彻落实科学发展观，坚持“四求先行”、“四用发展”要求，全面实施“以港兴市、工业强市”发展战略，立足扩大内需促进经济持续较快增长，着力改善民生，着力推进项目、品牌、创新和服务带动，着力转变发展方式，着力促进社会和谐，全面推进湄洲湾港口城市建设，为海西“两个先行区”建设作出新贡献。

　　经济社会发展的主要预期目标是：努力实现“十百千”目标，即财政总收入净增10亿元，生产总值净增100亿元，全社会固定资产投资净增100亿元，规模以上工业总产值超过1000亿元。全市生产总值增长13%；财政总收入增长19%，其中地方级财政收入增长19%；全社会固定资产投资额增长33.5%；工业增加值增长15%；社会消费品零售总额增长18%；外贸进出口总额增长10%；实际利用外资6亿美元（历史可比口径），增长7%；城镇登记失业率控制在3%以内；城镇居民人均可支配收入和农民人均纯收入分别增长8%左右；居民消费价格总水平涨幅控制在3%以内；人口自然增长率控制在7.5‰以内；单位生产总值能耗降低3%；二氧化硫排放量下降8%；化学需氧量排放减少0.5%。

　　今年要着力抓好以下十项工作：

　　（一）夯实“三农”基础，加速发展农村经济

　　继续深化农村改革。完善和稳定农村基本经营制度，保持土地承包关系稳定并长久不变。严格执行土地承包经营权流转的各项政策，推动农村土地依法、自愿、有偿流转，鼓励发展多种形式的适度规模经营。巩固扩大集体林权制度改革成果，完善林权、海域使用权抵押贷款等配套措施。创新农村工作机制，扎实推进“三百工程”，做好干部驻村工作。继续深化乡镇机构改革。健全农村民主管理制度。坚持最严格的耕地保护制度，节约集约用地，严格农村宅基地管理。促进城乡规划、产业布局、基础设施、公共服务、劳动就业一体化，统筹城乡经济社会发展。

　　积极发展现代农业。落实粮食生产各项政策，稳定粮食播种面积，推广优良品种，提高粮食总产量。继续调整农业结构，大力培育水产、水果、畜牧、蔬菜、食用菌、林业等主导产业，创建农产品优势产区，发展“一村一品”，推动农业产业化经营，壮大龙头企业。培育品牌农业，发展绿色食品和有机食品，加大农产品注册商标和地理标志产品保护力度，做好莆田荔枝、度尾文旦柚申报“国家地理标志产品保护”工作。推进农业标准化生产，争创一批国家级、省级农业标准化示范区和示范基地，建设一批农业技术推广中心。完善农产品质量安全检验检测体系。促进农业科技成果转化，推进农业机械化。健全良种繁育和动植物疫病防控体系。

　　改善农村基础设施。开展土地整理，建设3万亩高标准农田。完善农村水、电、路、沼气、通讯等基础设施，除险加固中小病险水库11座和病险海堤18.6公里，基本建成金钟水利枢纽、木兰溪霞林段防洪主体工程。争取木兰溪防洪工程三期、南北洋水系整治工程、金钟水利枢纽配套引水工程列入国家增投项目。新建一批渔港、海洋环境监测点、水产种质保护区、红树林保护区。继续改造农村电网。新增农村客运站（亭）200 座、农村水泥路300公里，改造农村危桥27座。新建农村沼气池8860口。加快城乡互联网建设，全市行政村以上区域实现互联网宽带接入全覆盖。

　　加大强农惠农力度。扩大公共财政对农村基础设施建设和公共事业发展的支持力度，推进农村扶贫开发，实施新的扶贫标准，帮助农村低收入群体脱贫致富。重点支持20个新农村试点村建设，挂钩帮扶20个革命老区村、民族村、边远山区海岛村、贫困村。“造福工程”搬迁4000人，地质灾害点搬迁588人。迁建秀屿村和罗屿村。继续实施库区移民后扶项目。

　　（二）坚持以港兴市，大力发展港口经济

　　切实加强港口规划。完成《莆田港总体规划》报批工作。深化“三湾”控制性规划，运用兴化湾南岸海湾数模、物模实验成果，加强兴化湾南岸岸线、前沿海域、后方陆域的规划管理。做好临港产业集中区、工业园区控制性规划，修编临港村庄整治规划，争取调整兴化湾南岸海洋功能区划，优化港口发展布局。

　　完善港口配套设施。续建秀屿和东吴作业区12个深水泊位、湄洲湾深水航道二期等港航项目，争取开工建设罗屿25万吨矿石码头、湄洲湾火电厂二期配套码头、林浆纸一体化5个配套码头。建成福厦铁路莆田段、莆田铁路站房、莆秀高速公路、仙港大道和城港大道一期。继续建设向莆铁路莆田段、湄洲湾港口铁路支线、福泉高速公路莆田段扩建工程、省道201线、涵港大道一期、仙游环山区公路。开工建设湄永高速公路莆田段、城港大道二期、石门澳路堤、沁峤路二期、东吴（罗屿）疏港公路、省道202线涵江段、省道306线拓宽改造工程和秀港路。抓紧做好兴化湾南岸港口铁路支线、湄洲湾至台中铁路轮渡、福广高速公路莆田段、兴化湾南岸至尤溪高速公路莆田段、湄洲岛跨海通道、莆田机场等重点项目前期工作。加大港区道路、通讯、供水、供电等基础设施投入，完善堆场、锚地等公共服务配套设施。

　　培育发展临港产业。认真落实加快发展港口经济30条意见，整合“三湾”资源，着力发展能源、石化、钢铁、船舶、林产加工、装备制造、港口物流等临港产业。加快建设LNG新增储罐、LNG冷能产业园、标准木业等重大临港工业项目，争取开工建设国航造修船基地、林浆纸一体化、石门澳临港产业园、国投湄洲湾配煤基地等重大临港工业项目，做好鞍钢冷轧、东吴石化基地、盘屿成品油储运基地、湄洲湾火电厂二期、莆田燃气电厂二期、莆田核电站、兴化湾南岸综合开发等重大项目前期工作。加快建设铁矿石、煤炭、木材、建材、食品等临港物流园区，努力打造区域性港口综合物流枢纽中心。扩建木材检验检疫除害处理区。

　　优化港口发展环境。拓宽港口建设融资渠道，鼓励各类投资主体参与港口开发与经营。深化港口管理体制改革，支持湄洲湾港口一体化管理体制改革。扩大港口设备投资，改善口岸联检条件，建立健全口岸查验协调机制，提高通关能力。完善港口应急救援预案，确保港口生产安全。

　　（三）建设工业强市，发展壮大工业经济

　　调整优化产业结构。认真落实加快工业发展30条意见，集中力量发展八大产业。石化产业要引进中上游产品项目，重点发展精细化工，延伸下游产业链。装备制造业坚持技术引进和自主创新相结合，加快发展以数控机床及铝铸件为主的专业设备及机械配套件。能源产业要加快气电、风电、火电、抽水蓄能电站建设。木材产业要重点发展科技含量、附加值高的深加工项目。电子产业要引导从加工装配向自主开发延伸，重点发展以小尺寸显示器为主的消费类电子产品及电子新元器件和新材料。鞋服、食品、工艺美术等传统优势产业要积极推进产品管理、品牌和营销创新，提升产业核心竞争力。

　　推进重点工业项目建设。确保开工建设闽中食品深加工、东峤风电场、新世纪覆铜箔板二期、华晶玻璃、绿森枇杷深加工等工业项目。力争开工建设光电伏特、丁基橡胶等生产性项目。跟踪服务66家重点企业和投资3000万元以上的新投产项目。推动佳通轮胎、众和纺织、三棵树涂料、万鑫金属、中科盛华、日晶玻璃、万邦电子、鼎盛五金、荣兴机械等企业增资扩产。巩固壮大规模以上企业，扶持规模以下企业，新增规模以上企业100家。

　　加快工业园区和集中区建设。进一步完善五个经济开发区、国家级木材贸易加工示范区和工业集中区基础设施。各级财政从今年起每年从经营性土地出让净收益中安排10%用于园区基础设施建设，园区内土地出让收益和园区内建设项目的城市基础设施配套费用于园区基础设施建设。继续清理工业项目闲置用地，盘活闲置厂房，改造“园中村”，建设标准化厂房，引导企业入驻。提高工业园区项目投资强度，提升园区产业集聚效益。

　　提高企业自主创新能力。滚动实施30个市级企业技术创新重点项目，创建13家省、市级企业技术中心，提升福建制鞋行业技术开发（莆田）基地建设水平。争创一批省级行业技术开发基地，建成省级木雕古典家具质检中心。筹建橡胶、鞋类工业信息中心。引导企业积极参与制定并采用行业、国家标准。充分利用“6？18”平台，嫁接一批技术创新项目。鼓励企业争创品牌，新创一批驰名商标、著名商标、名牌产品。

　　（四）扩大有效需求，抢抓持续发展先机

　　着力推进投资较快增长。落实积极的财政政策，优化投资结构，扩大投资规模。今年新安排192个在建重点项目、93个预备重点项目和57个前期重点项目，力争完成年度投资235.9亿元。根据中央和省新增投资政策方向，策划对接一批新项目，力争更多项目列入国家增投项目盘子。认真做好各类项目特别是工业项目的用地、环评、海域使用等前期工作，加快项目核准及建设进度，带动固定资产投资快速增长。

　　着力保持进出口持续增长。实施市场多元化战略，巩固传统出口市场，鼓励企业积极参加国内外各种展会，开拓新兴市场。落实支持外贸发展的政策，对大宗优势出口商品给予出口退税补贴，增加出口信用保险补贴和保单融资贴息。鼓励企业技术创新，提高商品档次和科技含量。完善中国鞋业出口基地建设，申报电子信息产品出口基地，创建天然气、橡胶、大豆和木材进出口基地。鼓励进口紧缺性资源性产品、原材料、先进技术和关键设备。

　　着力扩大消费需求。落实鼓励消费的政策，拓展消费领域，培育通信、旅游、休闲、文化、健身等消费热点，推动住房、汽车等大宗消费。完善城市商业网点，丰富商贸流通业态，提升城市消费水平。续建“万村千乡”市场工程，推动家电下乡，对农民购买指定的家电品种予以直接补贴，促进农村消费。加强社会信用体系建设，发展消费信贷。整顿规范市场秩序，加强食品药品安全管理，严厉打击制假售假、价格欺诈、坑蒙拐骗和危害人民群众健康的行为，保护消费者权益。

　　（五）加快发展服务业，进一步优化产业结构

　　促进现代服务业发展。加快建设各类物流园区，完善工艺美术城配套设施，开工建设涵江商贸物流园、仙游商贸中心区、荔城物流园区、秀屿物流商贸园区等项目，抓紧建设鞋材服装、珠宝首饰、油画等专业市场，跟踪服务天喔食品、双赢物流等项目，发展现代物流业。加快发展以软件、网络增值业务等为重点的信息服务业，规范发展会计、审计、法律、咨询、评估、设计、检测等专业服务业，培育发展创意文化等新兴服务业。培植总部经济、会展经济、休闲经济等现代服务业态。提升交通运输、邮政、商贸、文化娱乐、教育培训、社区服务业水平。

　　保持房地产市场持续健康发展。落实鼓励房地产发展的各项措施，科学合理供地，调整普通商品住房标准、税率和比例，降低住房交易税费。大力发展二手房和住房租赁市场，加大房地产金融信贷支持力度，提高公积金贷款标准。放宽二手房入户政策，实行外来人口购房市民待遇，防止房地产市场大起大落。加快建设安置房和廉租房，逐步改善城市中低收入家庭的住房条件。

　　加快旅游业发展。提升妈祖文化品牌，创建湄洲岛国家旅游度假区5A景区和工艺美术城、九鲤湖4A景区。整合旅游资源，完善景区交通网络，开发旅游新产品，发展妈祖朝圣、滨海休闲度假、工艺美术购物、生态农业、乡村、红色等特色旅游。加快建设一批星级酒店，提高旅游接待能力。

　　（六）建设宜居城市，不断完善配套功能

　　强化城市规划调控作用。完成新修编的城市总体规划报批工作。加快城市近期建设用地范围、工业园区的控规编制，深化交通、人防、抗震防灾、防洪排涝等专项规划。完成南北洋城市生态“绿心”和优秀近现代建筑保护利用规划，做好木兰溪两岸城市设计工作，塑造鲜明城市特色。做好湄洲岛城镇规划。

　　加强城市建设管理。围绕创建国家园林城市、省级文明城市目标，以承办第十四届省运会为契机，建成市体育馆等场馆。加快城市园林和公用设施建设，实现城建投资65亿元。建成莆田客运站。打通荔港大道、莆阳大道一期、文献路东扩、河滨路等市政道路。加快城市公共停车场、公共交通、路灯夜景、供电、通讯、给排水、环卫设施、城市燃气工程建设。继续推进护城河五期、顶墩、宫口、笏石、鲤中等片区改造。强化城市管理，提高市民素质，整顿交通秩序，美化市容市貌，提升城市形象。

　　加快妈祖城建设步伐。继续建设妈祖城核心区桥梁、道路等基础设施，完成年度投资6亿元以上。加快建设莆田一中妈祖城校区。争取开工建设新安国际医院。建设自来水、污水处理设施，筹建22万伏变电站。推进文甲码头步行街、忠门物流园区、山亭商贸城等项目前期工作。

（七）深化改革开放，提升开放型经济水平

　　提高内外资利用水平。积极引进外商、央企和省属企业，争取在临港产业、工业项目、基础设施和公用事业领域合作取得新突破。继续实施“民资回归”工程，为莆籍企业家回乡投资兴业提供更加优质的服务。围绕产业链招商，引进一批带动能力强的龙头骨干企业，延伸产业链，培育产业集群。充分发挥国家级LCD特色产业基地、国家级木材贸易加工示范区、中国鞋业出口基地、中国古典工艺家具之都、中国珠宝玉石首饰特色产业基地、中国银饰之乡等区域性品牌作用，采取“走出去、请进来”的办法，努力引进大项目好项目，促进产业升级。积极参加海峡两岸经贸交易会、中国福建商品交易会、福建省网上投资贸易洽谈会、中国国际投资贸易洽谈会等国内外知名展会。认真筹办第四届中国？莆田木业投资贸易洽谈会、第六届世界莆商大会、第四届海峡工艺品博览会、第十一届妈祖文化旅游节、第三届南北少林武术会演等重大节会活动。积极参加第四届兴安乡亲恳亲大会，吸引更多的海内外客商来莆投资兴业。

　　加强莆台港澳等地区经贸合作。充分发挥区位、港口和妈祖文化优势，提升莆台经贸、农业、旅游、文化交流合作层次。继续办好台湾农民创业园。加快湄洲岛对台客运码头配套设施建设，推动与台中港双向客运直航。启动秀屿港与金马澎货运直航。开展对台招商，邀请台湾重要工商团体和知名企业家来莆考察访问。加强莆港、莆澳交流与合作。落实侨务政策，凝聚侨心，发挥侨力，引进侨资侨智。积极参与闽东北协作区交流活动。筹建向莆铁路沿线城市联盟，充分发挥向莆铁路对我市港口经济的带动作用。

　　积极推进关键环节和重点领域改革。深化行政审批制度改革，进一步规范行政自由裁量权，清理审批事项，再造审批流程，推行网上审批，提高行政效率。完善国库集中支付和乡（镇）财县管改革，做好政府采购工作。推进水、电、成品油、药品、医疗服务等重要商品和服务的价格改革。培植一批龙头企业，扶持发展一批股份制企业，继续抓好企业改制上市工作。加强项目招投标监管，积极推行代建制。

　　（八）加强节能减排，促进生态文明建设

　　节约能源资源。修编第三次莆田市土地利用总体规划，强化建设用地监管，保护基本农田。加大海洋生态环境保护力度，合理开发海洋资源，节约集约使用海域和岸线。开展节水型社会试点建设，推进东圳等灌区节水改造，推广农业节水灌溉2万亩。继续实施“1520”节能示范工程，强化电力、化工、建材、轻纺、啤酒等高耗能行业的节能节水工作，做好资源综合利用和再生资源回收利用工作，提高工业固体废物和工业用水重复利用率。认真落实《公共机构节能条例》，政府机关要率先节约资源，优先采购和选用节能环保产品。

　　加强生态环境建设。坚持走生产发展、生活富裕、生态良好的文明发展道路，推进生态市建设。综合整治木兰溪、萩芦溪、南北洋水系等重要流域水环境，抓紧治理下磨溪，改善水生态环境。加快建立水源保护区管理长效机制和生态补偿机制，确保饮水安全。继续实施“农村家园清洁行动”，治理农业面源污染，推进农村生活垃圾集中处理。继续治理噪声和机动车尾气污染。加强矿山地质环境保护和治理，促进生态修复。继续建设沿海防护林、山区生态林，造林更新6万亩。支持建设湄洲岛国家级生态文明示范基地。

　　完善环保基础设施。继续整治工业污染，减少污染物排放。开工建设闽中污水处理厂二期和仙游、荔城、秀屿、北岸等四个工业园区污水处理厂及配套管网，续建霞林、下黄污水提升泵站，完善市、县区污水管网和污泥处理设施。推进市垃圾焚烧发电厂、医疗废弃物处理厂、仙游寨岭垃圾无害化处理场建设。扩建大湖垃圾处理场。建成一批山区、沿海垃圾焚烧炉和乡镇垃圾压缩转运站。建立海上船只溢油应急监测系统和应急小型设备库。

　　（九）强化财经工作，提高发展保障能力

　　培植发展财源。加强主体财源建设，重点支持一批科技含量高、辐射能力强并能形成产业链的重大工业项目，发展建筑业，扩大支柱财源。积极扶持农业产业化龙头企业，壮大基础财源。大力发展第三产业，落实促进旅游、会展、物流和商贸流通等服务业发展的政策，引进税收贡献率和附加值较高的服务型行业，增加第三产业的税收。逐步建立多层次、可持续的财源体系，优化财政收入结构，健全财政收入稳定增长机制。

　　强化收支管理。开展综合治税，加强税收政策宣传，提高全社会依法纳税意识。强化税源监控，抓好大宗税源、重点税种的征收工作。落实目标责任，完善考核机制，保证税款及时均衡足额入库。健全非税收入管理，加强行政事业单位资产管理。优化支出结构，科学合理使用资金，扩大资金运筹总量，提高资金使用效益。健全财政监督机制，严肃财经纪律。完善市对区财政体制，促进区域经济发展。

　　优化金融环境。认真落实中央适度宽松的货币政策，积极引导金融机构加大对重点工业项目、中小企业、节能减排、新农村建设、产业升级等重点领域和薄弱环节的信贷支持。深化农村信用合作社改革，创建莆田农村商业银行。推动邮政储蓄银行发展小额信用贷款，支持农村经济发展。适度发行地方债券，支持重大基础设施建设。积极推进企业与基金公司、财务公司、创业投资公司合作。推行债券融资、动产抵押、股权质押、商标质押等新型质押方式。扶持担保机构，新成立一批担保公司，逐步解决中小企业融资难问题。严厉打击逃废银行债务行为，确保金融安全。

　　（十）更加关注民生，促进社会和谐发展

　　坚持教育优先发展。合理调整城乡学校布局，整合优化农村教育资源，促进义务教育均衡发展。实施校舍抗震安全加固改造工程，加快农村中小学寄宿制学校建设。继续化解农村中小学债务。取消城市义务教育阶段借读费。更加重视学前教育，加快普及高中教育，大力发展职业教育，推进 4所国家级职业学校和2所省级职业学校实训基地和骨干专业建设。提高高等教育质量，全力办好莆田学院和湄洲湾职业技术学院。建成市教师进修学院。重视特殊教育，规范发展民办教育，大力发展远程教育和继续教育。全面实施素质教育，深化基础教育课程改革，提高教育教学质量。

　　加快发展科技文化体育事业。继续建设创新型城市，推进六大科技示范基地建设。广泛开展群众性精神文明创建活动，加强公民道德建设，提升文明程度。完善社区和乡村文化设施。建成莆仙大剧院和市博物馆，加快建设市艺术学校。组建福建省莆仙戏剧院和福建妈祖乐府。推进“妈祖信俗”、莆仙戏申报“世遗”工作，加大非物质文化遗产和文物保护力度。加强网吧等文化娱乐场所管理。健全全民健身服务体系，精心筹办第十四届省运会，促进竞技体育上水平。巩固广播电视“村村通”成果，推进电视数字化整体转换。发展哲学社会科学，繁荣文学艺术、广播电视、新闻出版事业。

　　提高人民群众健康水平。完善公共卫生体系，实施免疫规划，防治重点传染病和慢性病。开展爱国卫生运动，深化社区卫生服务。健全农村三级卫生服务网络，稳定农村医务人员队伍，完善新型农村合作医疗制度。建设市级新农合信息系统，实现农民就医“一卡通”。提升改造10个乡镇卫生院。加快莆田学院附属医院新区、市皮肤病防治医院等项目进度。加强人口与计划生育工作，完善计划生育家庭奖励扶助制度，扩大计划生育优质服务先进面，稳定低生育水平。着力抓好安全饮用水村村通等11件为民办实事项目。

　　努力扩大就业。完善创业服务体系，实施积极的就业政策，鼓励自主创业，以创业带动就业，新增就业3万人。组织实施各类职业培训，加大对被征地农民和零就业家庭等困难群体就业援助力度。高度重视高校毕业生就业和退伍转业军人安置工作。加强农民工就业技能培训，转移农村劳动力3.2万人。积极支持农民工返乡创业，从贷款发放、税费减免、工商登记、信息咨询等方面予以支持。

　　健全社会保障体系。完善城乡居民最低生活保障制度。扩大企业职工基本养老、医疗、工伤、生育和失业保险参保范围，逐步建立农村养老保险制度，建立农民工、被征地农民的养老保险制度。积极推进机关事业单位养老保险制度改革，完善城镇居民医疗保险工作机制。健全社会救助体系，发展慈善事业和社会福利事业。加快建设市慈康医院、市社会福利中心、市红十字备灾救灾中心和仙游县福利中心。新建扩建一批敬老院。重视发展老龄事业，保障妇女和未成年人合法权益，关心支持残疾人事业。落实低收入群体的补助办法，确保他们的生活水平不因物价波动而下降。

　　维护社会安定稳定。创建“平安莆田”，落实社会治安综合治理责任制，加强人防、物防、技防体系建设，严厉打击以“两抢一盗”为重点的刑事犯罪。认真做好信访工作，加大信访源头治理力度。进一步完善多元化矛盾纠纷调解衔接机制。加强“五五”普法，重视青少年法制教育。完善应急管理机制和预案体系，推进应急指挥平台建设，提高防灾减灾和处置突发事件的能力，保障社会公共安全。强化安全生产监督管理，深入开展“三合一”场所、烟花爆竹、道路交通、公众聚集场所等专项整治，有效遏制重特大安全事故。支持驻莆部队和武警部队建设，广泛开展“双拥共建”活动，巩固发展军政军民团结。高度重视保密工作，维护国家安全。继续加强统计、外事、气象、防震减灾等工作。

三、励精图治 有效作为 努力建设亲民为民政府

　　各位代表，全市人民对政府工作寄予殷切期望。我们一定要扎实推进政府自身建设，增强政府工作的原则性、系统性、预见性和创造性，提高推动科学发展的能力、构建和谐社会的能力、依法行政的能力，坚持为人民谋发展、为人民谋利益，励精图治，持续发展，在亲民为民上更有作为。

　　依法行政，在保障发展中更有作为。坚持向市人大及其常委会报告工作，执行人大及其常委会的决议，自觉接受人大及其常委会的法律监督、工作监督和政协的民主监督，认真办理人大代表议案、建议和政协提案。主动听取各民主党派、工商联和社会各界的意见和建议，支持工会、共青团、妇联等群众团体依照法律和各自章程开展工作。严格依照法定权限和程序行使权力，忠实履行职责。加强综合行政执法。强化行政监察、审计监督。推进电子政务和政府网站建设管理，深化政府信息公开。提高政府决策水平，始终把人民群众的愿望和要求作为决策的根本依据，在决策过程中务必通盘考虑和统筹协调各方利益，缓解不同社会群体之间矛盾，维护最广大人民群众利益。

　　重在运作，在推进发展中更有作为。为政之道，贵在实干，实干之道，重在运作。要用心运作，扎实运作，有效运作，推动发展。加强项目运作，立足长远策划生成项目，殚精竭虑沟通对接项目，攻坚克难推进落实项目，通过项目拉动投资增长，推动产业升级，带动实力壮大。加强政策对接，深入研究政策，把握政策，落实政策，用足用好中央和省扩大内需、促进经济增长的各项政策，认真贯彻执行我市加快港口、工业和农村经济发展等政策，努力把这些政策落实到每一层面、每一环节、每一个项目上，使各项政策发挥最大效应。加强市场运作，深化对社会主义市场经济规律认识，运用市场机制优化资源配置，加强招商引资、资源开发、城市经营等方面市场化运作，提高资源利用效益，增强经济发展活力。

　　勤政廉政，在服务发展中更有作为。各级政府和全体工作人员要强化服务意识，创新服务方式，提升服务水平。要敢作敢为，抓难碰硬，迎难而上，在发展中解决矛盾和问题，在解决矛盾和问题中推动发展。要求真务实，沉下心来干实事，扑下身子抓落实，真心真情关怀群众，用心用力支持企业，高质高效服务项目。要廉洁从政，各级领导干部肩负重责，手执公权，务必心有所畏、行有所止，务必清正为官、干净做事。推进机关效能建设，加强政府绩效管理工作。深入开展治理商业贿赂专项工作，纠正损害群众利益的不正之风，严肃查处各类违法违纪案件。同时，要宽容厚爱那些全心干事、踏实做事的干部，让广大干部放心放胆，敢闯敢干，一心一意谋事创业。

　　惠民利民，在和谐发展中更有作为。为民谋利高于一切，为民造福重于一切，为民服务大于一切。要从民心民意中谋求发展思路，从民智民情中汲取创新智慧，从民怨民忧中反省工作不足。时时为群众着想，处处替群众打算，时刻把人民群众的安危冷暖放在心上，真诚倾听群众呼声，真实反映群众意愿，真情关心群众疾苦，多为群众办好事、办实事，认真解决就业、就学、就医、住房、环保等事关人民群众切身利益的问题，切实保障人民经济、政治、文化、社会权益，促进社会和谐稳定。

　　各位代表！莆田是我们共同的家园，莆田的美好未来要靠全市人民共同创造。让我们在中共莆田市委的坚强领导下，以只争朝夕的精神、坚韧不拔的毅力、求真务实的作风，继续解放思想，坚持改革开放，推动科学发展，促进社会和谐，奋力开创湄洲湾港口城市建设新局面，以更加优异的成绩迎接建国60周年！
